# Assessing the risk of West Nile Virus seasonal outbreaks and its vector control in an urbanizing bird community: An integrative R_0_-modelling study in the city of Merida, Mexico

**DOI:** 10.1371/journal.pntd.0011340

**Published:** 2023-05-30

**Authors:** Alheli Flores-Ferrer, Gerardo Suzán, Etienne Waleckx, Sébastien Gourbière

**Affiliations:** 1 Departamento de Etología, Fauna Silvestre y Animales de Laboratorio, Facultad de Medicina Veterinaria y Zootecnia, Universidad Nacional Autónoma de México (UNAM), Ciudad de México, México; 2 International Joint Laboratory ELDORADO, IRD/UNAM, Mérida, Yucatán, México; 3 Institut de Recherche pour le Développement, UMR INTERTRYP IRD, CIRAD, Université de Montpellier, Montpellier, France; 4 Laboratorio de Parasitología, Centro de Investigaciones Regionales ‘Dr. Hideyo Noguchi’, Universidad Autónoma deYucatán, Mérida, Yucatán, México; 5 UMR5096 ‘Laboratoire Génome et Développement des Plantes’, Université de Perpignan Via Domitia, Perpignan, France; 6 School of Life Sciences, University of Sussex, Falmer, Brighton, United Kingdom; Oregon State University College of Veterinary Medicine, UNITED STATES

## Abstract

*Urbanization* is a global *trend* associated with key socio-economic issues, one of them being to control the transmission of infectious diseases to a urban fraction of the world’s population that shall reach 68% in 2050. While urban growth has been shown to favor mosquito species responsible for the transmission of the West Nile Virus (WNV), a major human arbovirosis, the effects of concomitant changes in the host bird communities remain hard to anticipate albeit essential to quantify disease risk and to plan control initiatives. We developed a R0 modelling of WNV transmission in a urban bird community to assess the risk of outbreak in Merida, one of the cities with the highest growth rate in Mexico. The model was parameterized using ecological and epidemiological data collected over the past 15-years on the local vector, *Culex quinquefasciatus*, and avian community. We identified a 3-weeks summer period during which the vector population strongly amplifies the WNV enzootic transmission and lead to a significant risk of outbreaks in humans. Extensive sensitivity analyses showed that urbanization induced changes in the bird community could lead to an up-to 6-fold increase in the duration of the risk period, while the daily risk could rise by 40%. Interestingly, the increase in *Quiscalus mexicanus* abundance had 4–5 times larger impact than any other change in the bird community. In such a context, annihilating the current and future risk of WNV outbreaks in Merida requires reducing the mosquito population by 13% and up to 56%, respectively. This study provides an integrative assessment of the current and future risks of WNV outbreak in the fast urbanizing city of Merida, and points toward the implementation of epidemiological monitoring combined with preemptive measures targeting both *C*. *quinquefasciatus* and *Q*. *mexicanus* populations, as they are expected to have synergistic effects.

## Author summary

*Urbanization* is a major trend in human evolution. By 2050, over 2/3 of the world population will live in urban areas. A key challenge associated with such a global trend is to protect urban residents against infectious diseases, while their environment may provide favourable conditions for parasites and other pathogens. The West Nile Virus (WNV) is a typical vector borne pathogen whose transmission to human is expected to be facilitated in urban environments, where mosquitoes transmitting the virus are favored. Here, we looked at the barely known effects of changes in the host bird community on the risk of WNV outbreak in humans. By designing empiricaly informed mathematical models of WNV transmission in the city of Merida, Mexico, we identified a 3-weeks summer period during which the virus circulation in urban birds lead to a risk of outbreaks in humans. Further analyses showed that changes in the bird community could increase the risk period up-to 6-fold and rise the daily risk by 40%. Such variations are primarily driven by the increase in a key reservoir species, *Quiscalus mexicanus*, whose effects are 4–5 times larger than those of other changes in the bird community. To control the current and future risks of WNV outbreaks in Merida requires reducing the mosquito population by 13% and up to 56%, respectively. These results strongly call for an epidemiological monitoring and preemptive measures targeting both *C*. *quinquefasciatus* and *Q*. *mexicanus* populations, as they are expected to have synergistic effects.

## Introduction

Urbanization is a paramount trend of our contemporary evolution. The fraction of the world’s population inhabiting urban areas is expected to rise from 55% in 2018 to 68% in 2050, with 90% of this increase taking place in Asia and Africa, while more than 70% of human beings already live in urban environments in Northern-America, Latin America and Europe [1]. Among the socio-economic challenges imposed by such a global change, it has been repeatedly warned that urbanization favors the emergence of new pathogens [2,3] and rises the burden of infectious diseases already afflicting human populations [4]. These global public health concerns are especially relevant for vector-borne diseases as the environmental changes associated with urbanization tend to increase vector abundance and species richness [5,6] as well as vectors contact with competent reservoirs [7–9] in inhabited and surrounding areas.

The West Nile virus (WNV) is a mosquito-borne pathogen that was first reported in 1937 in Africa [10]. Its worldwide spread has caused severe outbreaks in humans throughout North-America and Europe [11,12] and is presumably widely under-reported in South-America, where there is a need for both clinical and epidemiological studies to prevent it from becoming a major public health concern [13]. The virus is transmitted by *Culex spp*. and by secondary mosquito species of genera *Ochlerotatus*, *Aedes*, *Anopheles* and *Culiseta* [14,15] to vertebrate hosts that predominantly correspond to bird species, although 29 species of mammals, 3 species of reptiles and 1 species of amphibian were also found infected with WNV [16,17]. While in most non-avian host species, infected individuals can die of the infection [18,19], those species are typically considered as dead-end hosts for the WNV, so that the virus transmission primarily relies upon avian hosts [14]. A large set of previous studies has provided evidences that urbanization can alter the abundance and species structure of the mosquito community [20–23] and that urban landscapes create artificial habitats that serve as breeding sites for WNV-competent mosquito species, so that their frequency in the mosquito community can increase in such environments [24] and reference therein]. Although the contribution of the urban changes in the bird community to the risk of WNV outbreaks has been less investigated, there are nonetheless evidence that urbanization favors bird biodiversity reduction and species assemblage changes [25,26] with a correlated increase in Passeriformes and/or key species that represent competent reservoirs for the WNV [27].

The city of Merida in the Yucatan peninsula (Mexico) is a 1.12 million inhabitants urban center with an annual population growth rate of 2.5% over the last 40 years, which represents a fairly typical pattern in comparison with other intermediate Mexican cities [28]. This urbanization process has led the area covered by the city to increase by 300% between 1984 and 2018 [29], with significant land cover changes [29–31], an increase in mosquito breeding sites and a decrease in bird local biodiversity [32]. While there is still no documented outbreak of WNV infection in human across the entire Yucatan peninsula [13], evidences of birds’ infection by WNV have been collected since 2003 in the peninsula [33] and inside the city of Merida [34], indicative of an enzootic circulation of WNV. The urban sprawl and epidemiological situation encountered in Merida are likely to be illustrative of many urban places at risk of WNV outbreaks in Central and South America, and we thus aimed at providing a cost-efficient way to contribute evaluating such a risk, anticipating its potential evolution, and quantifying the efficacy of vector control interventions that would be necessary to eliminate the risk of transmission to human.

The complexity of a vector-borne pathogen’s transmission through a host community makes the use of mathematical models an essential approach to combine available data in the quantitative way required to identify the main determinants of transmission [35,36], predict the epidemiological outcomes of urbanization [37] and provide assistance to design effective control strategies [38–40]. In this study, we identify the network of WNV transmission in the city of Merida from ecological studies of the urban bird community, molecular identification of *Culex quinquefasciatus* blood meals and from experimental studies of the competences for WNV of the passeriform and columbiform species that shape this host community. We then model WNV transmission in such an urban bird community and use a next generation matrix approach to derive a formal expression of the WNV R_0_ in this context. These modelling and eco-epidemiological data provide key estimates and insights into i) the current risk of WNV outbreaks, ii) its expected variations with changes in the composition of the host community associated with urbanization, and iii) the level of vector control that could annihilate the risk of outbreaks of infection in humans.

## Materials and methods

### Modelling West Nile virus transmission in a urban bird community

We developed a R_0_ model to assess WNV transmission and the risk of outbreak in the urban area of Merida that is populated by a reservoir bird community made of passeriform and columbiform species. Our modelling account for vector-borne transmission by the dominant vector species, *Culex quinquefasciatus*, between several groups of bird species defined with respect to their local abundances and reservoir competences. The R_0_ was calculated from the corresponding community graph using the Next Generation Matrix (NGM) method, as previously done for other vector-borne pathogens circulating in host communities [36,41–43]. The general expression of R_0_ for the WNV transmitted by 1 vector to N groups of bird species is derived below, and subsequently tailored according to the specific entomological and ornithological situation encountered in the urban environment of Merida (see section ‘Characterization of the local network of transmission’).

The NGM is typically defined from two types of quantities; the mean number of hosts of group j that are infected by one infected vector during its infectious lifetime (k_jV_) and the mean number of vectors infected by one infected host of group j during its infectious lifetime (k_Vj_).

The expected number of birds of group j infected by one newly infected mosquito (k_jV_) is given by the product of i) the probability that the infected mosquito survives the extrinsic incubation period to become infectious, ii) the daily number of bites made by an infectious mosquito (a), iii) the probability that such a bite is made on a host of group j (p_j_), iv) the per bite probability of (vector to host) virus transmission (b), and v) the duration of the mosquito infectious lifetime. The mosquito survival probability to the extrinsic incubation period (i) was calculated by assuming a constant incubation rate (κ) and a constant vector death (μ_V_), so that this probability (that incubation ends before death) equals (κ/(κ + μ_V_)). Further assuming that an infectious mosquito will remain so during its lifetime, the duration of the mosquito infectious lifetime (v) is given by 1/μ_V_. Taken together this leads to:

$$k_{jV}=\frac{\kappa ap_{j}b}{\mu_{V}(\kappa +\mu_{V})}$$ (1)

The mean number of vectors infected by one infected host of group j during its infectious lifetime (k_Vj_) is obtained by multiplying i) the average number of mosquito per host (m), ii) the mosquito feeding preference for hosts of group j (p_j_), iii) the daily number of bites made per mosquito (a), iv) the per bite probability of (host j to vector) virus transmission (c_j_), and v) the duration of the host infectious lifetime. We assumed that an infectious host will remain so until it dies because of WNV induced (α) or intrinsic (μ_j_) mortality. The duration of the infectious lifetime for a host of group j is then given by 1/(μ_j_+α). Taken together this leads to;

$$k_{Vj}=\frac{mp_{j}ac_{j}}{\left(\mu_{j}+\alpha \right)}$$ (2)

The R_0_ for the WNV transmitted by 1 vector to N groups of bird species can then be calculated as the dominant eigenvalue of the NGM:

$$NGM=(\begin{array}{ccccc} 0 & \cdots & k_{\mathrm{Vj}} & \ldots & k_{\mathrm{VN}}\\ \vdots & \ddots & \vdots & 1 & \vdots \\ k_{\mathrm{jV}} & \ldots & 0 & \ldots & 0\\ \vdots & 0 & \vdots & \ddots & \vdots \\ k_{\mathrm{NV}} & \ldots & 0 & \ldots & 0 \end{array})$$ (3)

where k_Vj_ and k_jV_ are defined as explained above.

This eigenvalue can be expressed as:

$$R_{0}=\sqrt{\sum_{j=1}^{N}k_{jV}k_{Vj}}=\sqrt{\sum_{j=1}^{N}{\left(ap_{j}\right)}^{2}m\frac{bc_{j}\kappa }{\mu_{V}(\kappa +\mu_{V})(\mu_{j}+\alpha )}}$$ (4)

Such expression of R_0_ allows predicting when, i.e., for which set of parameter values, the pathogen is expected to circulate within the modelled host community. We will use the condition R_0_>1 to indicate when the WNV is able to circulate among the urban reservoir host community, which is typically thought as an estimate of the local risk of spillover to humans, e.g. [44,45].

### Model parameterization to the local passeriform and columbiform community and the transmission of WNV by *Culex quinquefasciatus* in Merida, Mexico

The above general expression of the NGM was tailored to describe the transmission of WNV in the urban area of Merida and R_0_ was used as a measure of transmission risk to humans. Using data that have been published over the last 15 years, we identified the local network of transmission that set the dimension of our model and we derived independent estimates of all quantities defining the corresponding k_jV_ and k_Vj_.

### Characterization of the local network of transmission

A local network of transmission is defined according to the set of local reservoir hosts that belong to the vector feeding range and with respect to their levels of competences for WNV. To characterize this network in the urban environment of Merida, we combined local field studies of i) the bloodmeals of *Culex quinquefasciatus*, ii) the species composition of the bird community and iii) their competences for WNV. While 10–15 mosquito species have been reported in Merida [46,47], only two collected species were known to be capable of transmitting the WNV, *C*. *quinquefasciatus* and *C*. *thriambus* [47], with evidences of WNV infection found only in the former in Mexico [47]. In addition, *C*. *quinquefasciatus* was shown to represent 88.7% of mosquitoes collected in the city of Merida [48], so that the general modelling proposed for the transmission of the WNV by 1 vector to N groups of bird species does fit adequately the local situation encountered in the city of Merida.

*Bloodmeal analysis of C*. *quinquefasciatus*. The local vertebrate host community on which *C*. *quinquefasciatus* feed on in Merida was characterized from a bloodmeal analysis performed on 240 engorged mosquitoes collected in the backyards of 40 houses in Merida from January 2005 to December 2005, using resting wooden boxes [49]. This study showed that vectors typically feed on birds from three orders; Galliformes (47.1%), Passeriformes (23.8%) and Columbiformes (11.2%), on domesticated mammals; dogs (8.8%), cats (1.2%), horses (0.8%) and pigs (0.4%), as well as on humans (6.7%). While mammals and Galliformes are considered as not competent for WNV [14,50–54], the high proportion of meals made on passeriform and columbiform species that are typical bird reservoirs [14,55,56] and the concomitant frequency of bloodmeals on humans, confirm the existence of a transmission path that could potentialy results in local outbreaks of WNV. To fine-tune the definition of the local network of transmission and assess such a risk, we identified the passeriform and columbiform species present in Merida from a local field study and derived estimates of their competences for WNV from the literature.

*Passeriform and columbiform host species and their WNV competences*. A census of the bird community performed between October 2004 and September 2005 in parks of the city of Merida reported 114 species from an overall collection of 9049 individuals [57]. A total of 65 passeriform species belonging to 15 families were found, 4 of which being considered as non-resident migrating species that we removed from our analysis. The *Quiscalus mexicanus* species was largely dominant, corresponding to 39.9% of all catched Passeriformes, each of the other species representing between 0.02% and 5.9% of the census (see S1 Appendix). A total of 6 columbiform species were found, with *Zenaida asiatiaca* representing up to 76.5% of the catched Columbiformes while the relative abundance of other species varied between 0.04% and 9.6% (see S1 Appendix). Independent predictions of the local bird community based on sampling along transects located in 4 different sites located inside Merida suggested that this census reported 80%-95% (ACE) and 79%-94% (Chao1) of the expected bird species richness [57]. We then looked into the litterature for estimates of the reservoir competence index (C) for each of those 67 passeriform and columbiform species, which came from [58] for *Q*. *mexicanus*, and from [55,59] for all other bird species (see S1 Appendix). When such estimates were not available for the reported species, we used estimates gathered for the closest relatives according to the phylogenetic tree provided in [60]. One of the 61 resident passeriform species was not present in this phylogeny and was removed from the analysis as it accounted for only 0.13% of the collected individuals. The 60 remaining passeriform species (see S1 Appendix) were then partitioned into four categories according to their reservoir capacities, with groups P_1_ to P_4_ defined from conditions 0<C≤0.5, 0.5<C≤1; 1<C≤1.5 and C>1.5. The resulting groups were made of 19, 32, 0 and 8 species, respectively, so that we ultimately considered the first two groups, and merged group 3 and 4 into a third group where C>1. We decided to single out the *Q*. *mexicanus* species since it is well recognized to be the most abundant passeriform species [57] and a highly competent reservoir [58], whose abundance is expected to keep rising with further urbanization. As they were no variations between competence estimates among Columbiformes (see S1 Appendix), we considered them as a single group.

**Fig 1:**
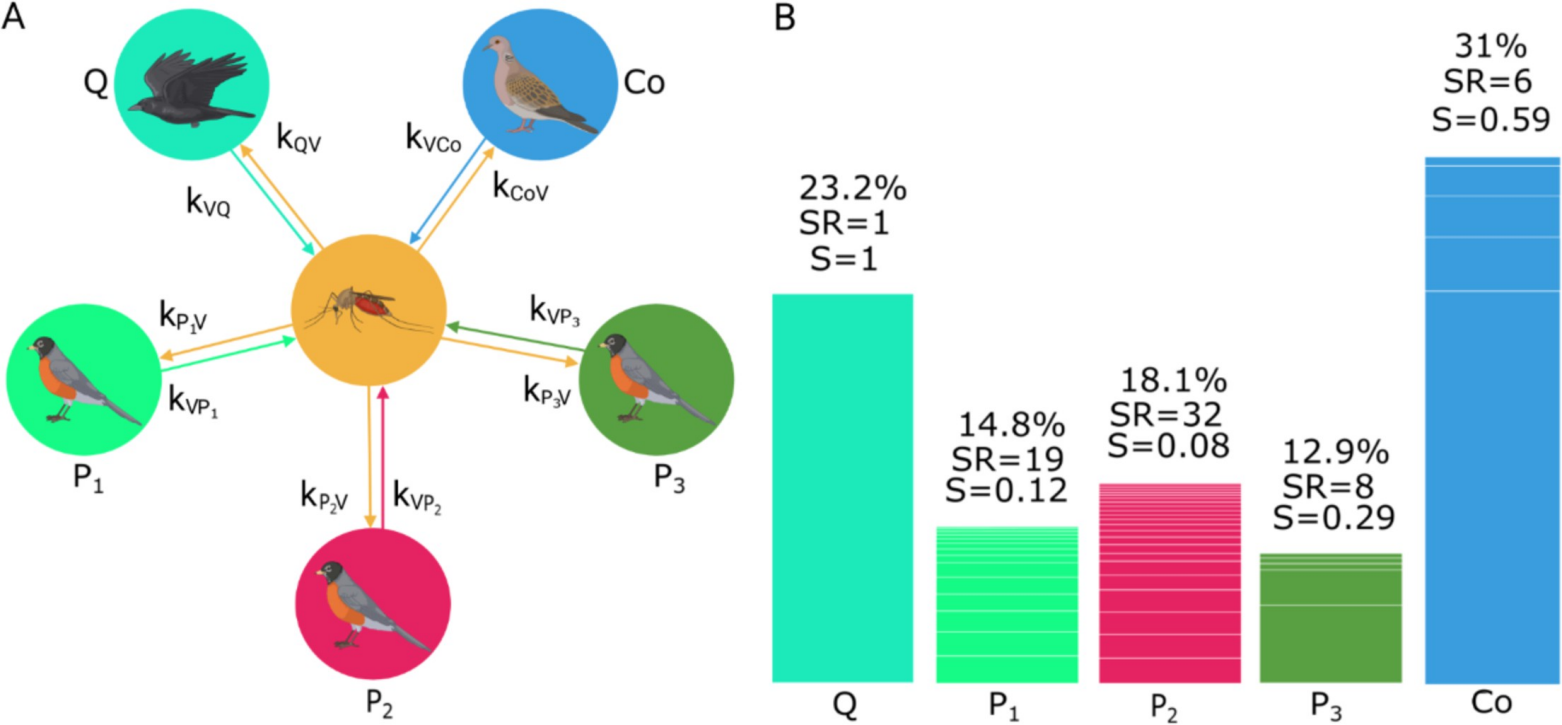
The network of West Nile Virus transmission in Merida. (**A**) Community graph with the vector *Culex quinquefasciatus*, and the 5 groups of host species; *Quiscalus mexicanus* (Q), the three groups of passeriform species with reservoir capacities 0<C≤0.5 (P_1_), 0.5<C≤1 (P_2_) and C>1.0 (P_3_), and the group made of all columbiform species (Co). The proportion of individuals that was found to belong to each of these 5 groups in the local bird host community and the species richness (‘SR’) and Simpson’s diversity (‘S’) of each host groups are shown in (**B**). The white lines represent the proportions of the different species within each group. Created with BioRender.com.

*The network of WNV transmission*. The network of WNV transmission in Merida was thus described as made of a locally dominant vector species, *C*. *quinquefasciatus*, and the above N = 5 groups of reservoir birds that we thereafter refer to by using the following set of index J = {Q, P_1_, P_2_, P_3_, Co}, with (Q) standing for *Q*. *mexicanus* and (Co) for all other columbiform species. Fig 1 shows the community graph corresponding to this network of transmission, along with the part of the bird community that each of the 5 groups of hosts represent, their species richness and diversity measured using Simpson index.

### Host and vector parameter estimates

To assess the transmission of WNV in this network, we estimated all parameters involved in the expression of R_0_ established in section ‘Modelling West Nile virus transmission in a urban bird community’ (Eqs 1–4). A description of the derivation of those estimates is provided below, and Table 1 summarizes the quantitative values taken by all parameters defining R_0_.

**Table 1:** Definition and estimates of the model parameters.

	Symbol (unit)	Estimate	References
***Vector feeding***			
Biting rate	a (days^-1^)	0.25^(a)^	[48]
Feeding preferences	*p*_*j*_^(1)^	0.22 for passeriform species (including *Q*. *Mexicanus*) 0.12 for columbiform species	[48]
***Vector and host abundance***			
Vector abundance	N_V_^(2)^	98,136,160,147,54,78,108,267,99,111,265,95	[49]
Host abundance	N_H_^(2)^	861, 669, 758, 715, 684, 589, 469, 804, 602, 605, 519, 739	[57]
Vector to host ratio	m^(3)^	11, 19.6, 20.3, 19.8,7.6,12.7,22.2, 32, 15.8, 17.7, 49.2, 12.4	
***Vector and host mortality***			
Vector death rate	*μ*_*V*_ (days^-1^)	0.08 (rainy) 0.22 (dry) ^(b)^	[48]
Host death rate	*μ*_*j*_^(1)^ (days^-1^)	{2.19, 3.2, 3.1, 2.6, 1.5} x 10^−4 (c)^	[62]
***Probabilities of WNV transmission***			
Compounded per bite probabilities of transmission^(4)^	b *c*_*j*_^(1)^	0.545, 0.36, 0.11, 0.016, 0.36	[55] [58] [59]
***WNV life-history***			
Incubation rate in vector	κ (days^-1^)	0.106 ^(d)^	[64]
WNV-induced host death rate	α (days^-1^)	0.25 ^(e)^	[58]

^(1)^ j takes value in J = {Q, P_1_, P_2_, P_3_, Co}, with (Q) standing for *Quiscalus mexicanus*, (P_1_, P_2_, P_3_) for other passeriform species and (Co) for Columbiformes. ^(2)^ Both vector and host abundances were estimated monthly, and are given from January to December. ^(3)^ The vector to host ratio was calculated after the field estimates of N_V_ were corrected by the ratio A_M_/A_v_ to account for the sampling area and by the efficacy of traps estimated to be around 70% (Garcia-Rejón J. and Baak-Baak C, personnal communication). ^(4)^ Only the product of the per bite probability of (vector to host) virus transmission, i.e. parameter b, and of the per bite probability of (host j to vector) virus transmission, i.e. parameters c_j_, are relevant to the calculation of R_0_ and were estimated as explained in the main text. ^(a)^ This biting rate estimate implies that mosquitoes bite on average once every 4 days (1/0.25). ^(b)^ These vector death rates correspond to an (adult) mosquito life expectancy of 12.5 (1/0.08) and ~4.5 (1/0.22) days during rainy (May-October) and dry (November-April) seasons, respectively. ^(c)^ These host death rates are equivalent to life expectancies of ~12.5 (1/(2.19x10^-4^x365), ~8.5 (1/(3.2x10^-4^x365), ~8.8 (1/(3.1x10^-4^x365), ~10.5 (1/(2.6x10^-4^x365) and ~18.2 (1/(1.5x10^-4^x365) years, respectively. ^(d)^ This incubation rate corresponds to an incubation time of ~9.4 days (1/0.106). ^(e)^ This additional host death rate leads infected hosts to die after 4 days (1/0.25).

*Vector feeding rate (a) and preferences (p*_*j*_*)*. The overall biting rate of *C*. *quinquefasciatus* was estimated by [48] and found equal to 0.25 bite a day, which is consistent with other estimates derived in [61]. Preferences were derived from the study by [49] that provided the percentage of bites made on Passeriformes, including *Q*. *Mexicanus*, and Columbiformes and were used for each of the corresponding groups.

*Death rates of host (μ*_*j*_*) and vector (μ*_*V*_*)*. Death rates of vector and hosts were estimated using the typical assumption that their lifetime follows an exponential distribution with a mean equal to the average life duration expectancy. The average species lifespan was found in [48] for *C*. *quinquefasciatus*, in [62] for 44 other hosts species, and in 4 open-access scientific databases visited in June 2021 for the last 16 species (see S1 Appendix). For each of the five groups of hosts, the death rate was then calculated as the average of those species’ death rates weighted by their relative abundance. As strong differences were reported in *C*. *quinquefasciatus* survival between the rainy and dry seasons [48], we considered two separate estimates of the vector death rate for the corresponding period of time, i.e. May-October and November-April, respectively.

*Vector/host ratio (m)*. The mosquito to host ratio was calculated from abundance estimates derived from two longitudinal field studies on vector [49] and hosts [57] in the urban environment of Merida. The vector abundance was estimated from [49], where mosquitoes were trapped from 40 backyards, the corresponding backyards and their immediate neighbourhood. The vector sampling area (A_V_) was considered to be proportional to the mosquito dispersal range, which was calculated as the surface of a circle with a radius equal to the mean dispersal distance of *C*. *quinquefasciatus*, i.e. 0.27 km per day [63]. Vector abundance was then calculated by scaling up from the sampling area (A_v_ = 9.16km^2^) to the total area of Merida (A_M_ = 883.4km^2^). Importantly, these field estimates of the vector population were made on a monthly basis. As [63] also provided monthly variation of the total host abundance, we could estimate the vector to host ratio for each month of the year. The ratio was found to vary between 7.6 and 49.2, consistent with previously estimated values that were found in the range of 10–100 vectors per host [36,64].

*Per bite probabilities of transmission (b*, *c*_*j*_*)*. A complete set of independent estimates of those probabilities is arguably the largest difficulty in calculating the R_0_ for a multiple hosts network of transmission. It is however important to note that only the products of those probabilities are relevant to the calculation of R_0_. Now, those products (bc_j_) can readily be estimated from the ratio C/D, where C stands for the host reservoir competence defined in [54] and D represents the duration of viremia in days, respectively. We calculated those quantities from bird species competence estimates already used to characterize the local network of transmission and from the duration of infection found in [55,58,59] (see S1 Appendix). Those products ranged from 0.11 to 0.545, similar to the range of values that were obtained in other modelling studies of WNV transmission, e.g. [36,64,65].

*WNV life history*. The duration of the extrinsic incubation period was calculated from the rate of incubation estimated by [64], and found equal to 9.4 days, which is very similar to previous estimates found in *C*. *quinquefasciatus* and other mosquito species [36] and leads to an incubation rate in vector κ equals to 0.106. The WNV induced host death rate was estimated under the same assumption as the natural host death rates (see above) and from infection experiments that provided life duration expectancy upon infection for *Q*. *mexicanus* and other passeriform and columbiform species [58].

### Analysing the risk of West Nile Virus outbreak in the urban area of Merida

#### Seasonal variations of the risk of WNV outbreak

We started our analysis by integrating all the estimates provided in Table 1 into the calculation of R_0_ for each month of the year. The observed temporal variations in the vector death rate and in the vector to host ratio are indeed likely to lead to marked seasonal variations of the risk of WNV outbreak, which obviously is a key public health information to anticipate. From the predicted variations we then calculated i) the annual period of time when R_0_ exceeds 1, ii) the average value of R_0_ during such a period of risk of WNV spill-over to humans, and iii) the maximal value of R_0_, thereafter denoted as P, $\bar{R_{0}}$, and $R_{0}^{max}$, respectively.

#### Systematic sensitivity analyses of the risk of WNV outbreak

We completed our situational analysis by performing systematic sensitivity analyses of the predicted seasonal variations of the risk of WNV transmission to changes in each of the model parameters. We then increased or decreased the estimated values model parameters appearing in Table 1 by 5%, 10%, 15%, 20% and 25% and we measured the corresponding P, $\bar{R_{0}}$ and $R_{0}^{max}$. Such analyses were intended not only to provide a comprehensive view of the potential consequences of the inevitable uncertainty in the estimates of parameters appearing in Table 1, but also to identify the parameters whose future changes could have the most significant impact on the risk of WNV outbreak.

### Changes in bird reservoir community associated with urbanization and their implications for the risk of WNV outbreak

With the increase of human population in Merida and the associated changes in the urban environment, one expects a progressive enrichment of the bird community with the more synanthropic species [66]. We simulated the most likely scenario whereby an increase of the abundance of *Q*. *mexicanus* and/or Columbiformes is compensated by a proportional reduction in the rest of the bird community. The impact of such changes on the risk of WNV outbreaks was assessed while the abundance of *Q*. *mexicanus* and/or Columbiformes were progressively increased from 0 to 50% by 10%. We systematically measured the resulting changes in P, $\bar{R_{0}}$ and $R_{0}^{max}$. All those variations were compared through elasticity measures that provided the proportional changes in P, $\bar{R_{0}}$, $R_{0}^{max}$ for a proportional change in the abundance of *Q*. *mexicanus* and in the abundance of Columbiformes, i.e. the percentage of variations in P, $\bar{R_{0}}$ and $R_{0}^{max}$ when the abundance of *Q*. *mexicanus* or the abundance of columbiforms is increased by 1%. A value of elasticity was calculated to assess the response to a change in *Q*. *mexicanus* provided that the abundance of columbiforms has already raised by 0%, 10%, 20%, 30%, 40% and 50%, and the same procedure was applied to evaluate the impact of changes in the abundance of columbiforms, when the abundance of *Q*. *mexicanus* has already raised by similar amounts, i.e. between 0% and 50% by 10%.

### Potential of vector control to limit the risk of WNV outbreak

To complete the above analyses, we aimed at exploring how efficient should vector control be for the risk of WNV outbreaks to be annihilated. We estimated the fraction of the vector population that should be removed for R_0_ to remain lower than one all year round, and we did so for the current bird community as well as for the various alternative compositions of this community considered when assessing the impact of urbanization.

## Results

### What is the current risk of WNV outbreak in Merida, Mexico

**Fig 2:**
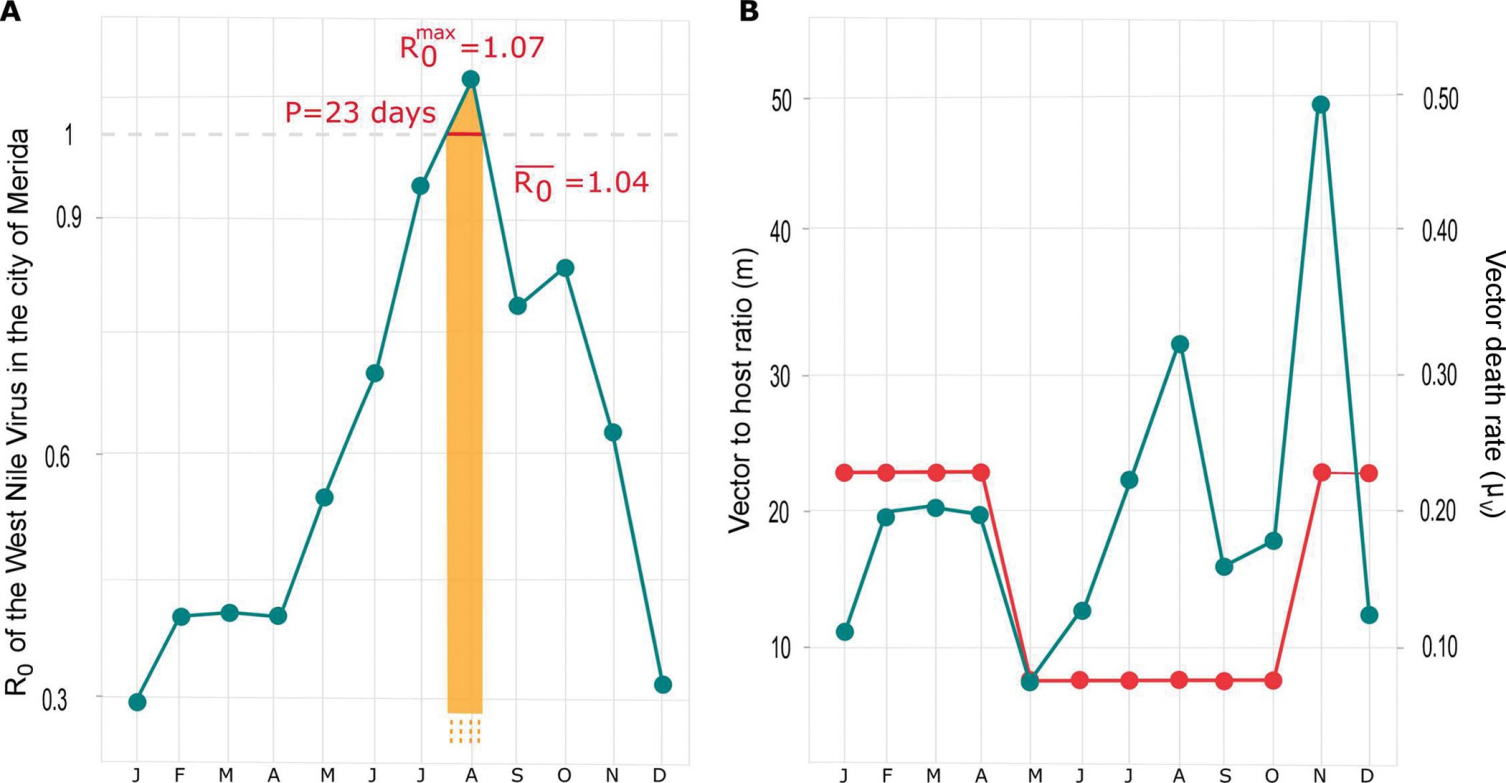
Seasonal variations of the risk of West Nile Virus outbreak in Merida, Mexico. (A) The monthly values of R_0_ show a strong temporal pattern including a period of P = 23 days with a risk of outbreak (R_0_>1) and a maximal risk in August equals to $R_{0}^{max}$ = 1.07. The average value of R_0_ during the identified period of risk of WNV spill-over to humans was found to be $\bar{R_{0}}$ = 1.04. (B) The seasonal variations in R_0_ are induced by the changes in the vector to host ratio (m) and in the vector death rate (μ_V_) estimated from [44], [52] and [56] appear in green and red, respectively.

The R_0_ values estimated from the parameterized network of transmission show a strong seasonal pattern with WNV circulation amongst urban reservoirs peaking in July and August, and being at its lowest level between mid-November and mid-April (Fig 2A). The season with a maximal risk of WNV outbreak corresponds to the period of time when, concomitantly, the vector to host ratio (m) is at (one of its) highest and the mosquito death rate (μ_V_) is at its lowest (Fig 2B), as expected from the general expression of R_0_ provided by Eq 4. The value of R_0_ reaches a maximum value $R_{0}^{max}$ = 1.07 at the beginning of August, indicating that there is, at that time, a sustained WNV transmission between birds in Merida and a risk of outbreak of human infections. This risk seems to be restricted in time as R_0_ stays above one for a period of P = 23 days, from mid-July to the end of the first week of August, when it is on average equals to $\bar{R_{0}}$ = 1.04. Interestingly, all the above predictions were shown to be robust to the inclusion of a bird recovery in our modelling, as $R_{0}^{max}$, P and $\bar{R_{0}}$ were then changed by a maximum of -3.6%, -17% and -1.9%, respectively (see S2 Appendix).

### Sensitivity analyses of the intensity and duration of the risk of WNV outbreak in Merida, Mexico

**Fig 3:**
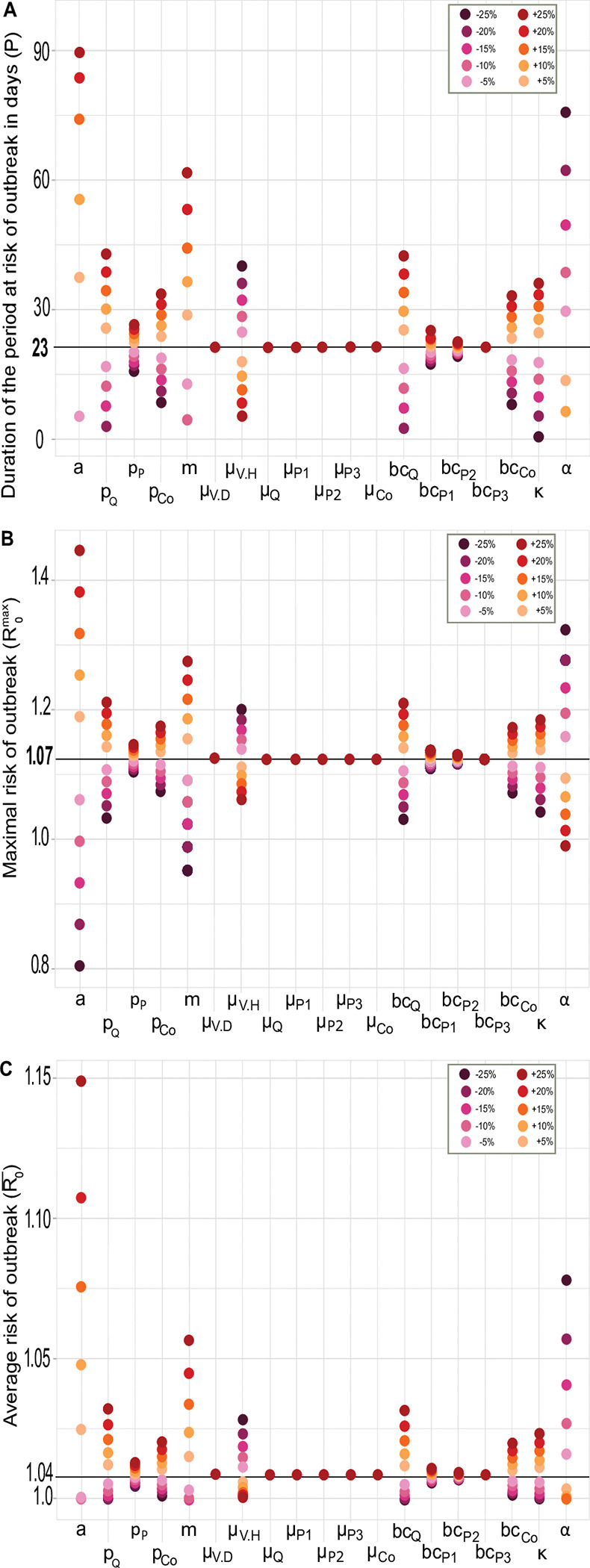
Sensitivity analyses of the risk of West Nile Virus outbreak in Merida, Mexico. The effects of -25% to +25% changes in each of the model parameters value are shown for the (**A**) duration of the period at risk of WNV outbreak (P), and the (**B**) maximal ($R_{0}^{max}$) and (**C**) average ($\bar{R_{0}}$) values of such a risk. The reference values of P, $R_{0}^{max}$ and $\bar{R_{0}}$ that are shown in bold and represented by horizonal lines in **A**-**C** are those obtained from the model parameters estimates given in Table 1 and that appeared in Fig 2.

The general pattern of seasonal variations shown in Fig 2 is very robust to all changes we made to all model parameters (see S3 Appendix). We thus focused our sensitivity analysis on the three key summary quantities; the amount of time P where R_0_>1, the average risk of WNV outbreak during such a period, $\bar{R_{0}}$, and its maximal value $R_{0}^{max}$. As expected, all of them increase in value with most of the parameters that contribute positively to the analytical expression of R_0_ provided by Eq 4. Those parameters can easily be anticipated from this expression and our sensitivity analysis consistently shows that the vector biting rate (a), the vector preferences for each (competent) bird species (all p_j_’s), the vector to host ratio (m), the probabilities of transmission (all bc_j_’s but bc_P3_ that has no effect) and the rate of WNV incubation in vector (κ) contribute to increase the risk period and the maximal and average levels of risk (Fig 3). On the contrary, larger vector (*μ*_*V*_) and WNV-induced host death rate (α) lower the duration and the risk of transmission, as expected from their contribution to the expression of R_0_. Meanwhile, host mortalities (*μ*_*j*_) did not show any of the negative effects that were expected from Eq 4. While the identified (positive or negative) effects of these various parameters on P, $\bar{R_{0}}$ and $R_{0}^{max}$ make epidemiological sense, our modelling further allowed to quantify the extent to which they affect the risk of WNV outbreaks (Fig 3). The larger variations in the three above key quantities were induced by the vector biting rate (a), the vector to host ratio (m) and the WNV-induced host death rate (α). Increasing the vector biting rate by up to 25% led to a 3.8 times broader period at risk and simultaneously raised the maximal and average risk by 36% and 10%. A decrease in the WNV-induced host death rate extended by up to 3.3 times the duration of the risk period, while the maximal and average risk increased by up to 24% and 3%. Similarly, an increase in the vector to host ratio produced 2.7 times longer risk period with a maximal and average risks increased by 20% and 3%, respectively. The next two parameters with the most significant impacts were both associated with *Q*. *mexicanus*. The vector preference for *Q*. *mexicanus* (p_Q_) had 3 to 5.2 more impact on the duration of the risk period and the maximal and average risks during this period, than the preference for other Passeriformes and Columbiformes. Meanwhile, the probability of WNV transmission associated to *Q*. *mexicanus* had 3.7 to 8.7 more effects on those same three quantities than the probabilities associated with other Passeriformes and Columbiformes. Finally, although a decrease in the vector mortality rate during rainy season also contributed to broaden the period and (maximal and average) risks of transmission of WNV in Merida, the vector mortality during the dry season and the host mortality rates had virtually no effect on any of these three predicted quantities.

### Urbanization, change in the composition of the bird community and its implications for the risk of WNV outbreak in Merida, Mexico

**Fig 4:**
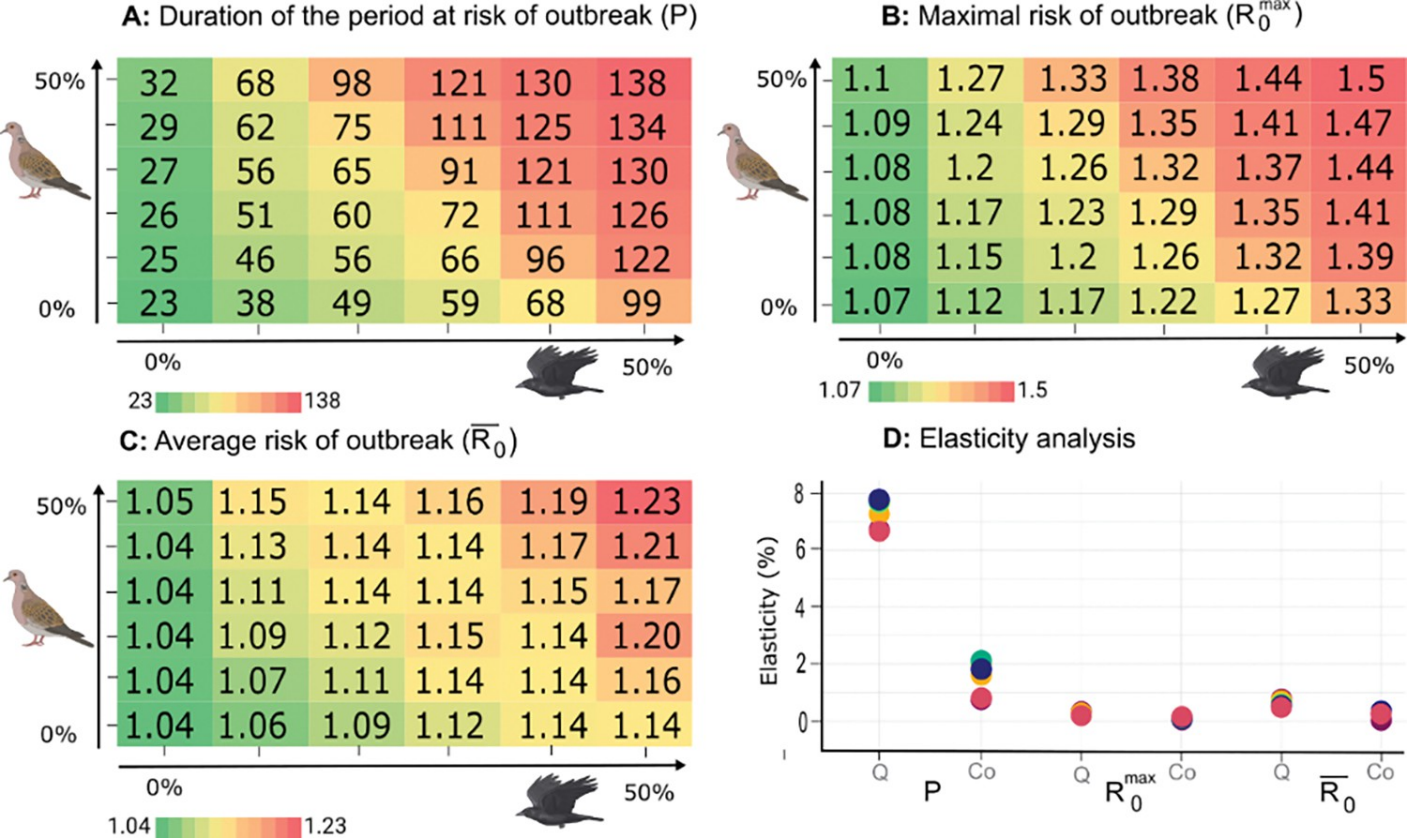
Changes in the bird community associated with urbanization and its implications for the risk of West Nile Virus outbreak in Merida, Mexico. The effects of increases in the abundance of *Quiscalus mexicanus* (along the x-axis) and/or columbiform (along the y-axis) species are shown for the (**A**) duration of the period at risk of WNV outbreak (P), and the (**B**) maximal ($R_{0}^{max}$) and (**C**) average ($\bar{R_{0}}$) values of such a risk, whose reference values appear in bold in **A**-**C**. The elasticity measures shown in (**D**) represent the percentage of growth in P, $R_{0}^{max}$ and $\bar{R_{0}}$ when the abundance of *Q*. *mexicanus (Q)* or the abundance of columbiforms (Co) is increased by 1%. An elasticity measure was calculated to assess the response to a change in *Q*. *mexicanus* provided that the abundance of columbiforms has already raised by 0%, 10%, 20%, 30%, 40% and 50%, which led to 6 different elasticity values shown as colored circles where green = 0%, violet = 10%, yellow = 20%, light green = 30%, red = 40% and blue = 50%. The same procedure was applied to evaluate the impact of changes in the abundance of columbiforms, when the abundance of *Q*. *mexicanus* has already raised by similar amounts, i.e. from 0% to 50% by 10%, and using the same key for colors. Created with BioRender.com.

While the above sensitivity analyses allowed to quantify the effects that independent changes in each of the model parameters have on the risk of WNV outbreaks, the modification of the bird community associated with the development of a city such as Merida will concomitantly involve several of these effects. We assessed their overall outcomes on P, $\bar{R_{0}}$ and $R_{0}^{max}$ (Fig 4A–4C). The amount of time where people are exposed to a risk of WNV outbreak (P) increases steadily from 23 to a maximal value of 138 days when the host community was enriched in *Q*. *mexicanus* and Columbiformes. The average and maximum value of the risk also rise when *Q*. *mexicanus* and Columbiformes are made more abundant, but in substantially lower amount. The value of $R_{0}^{max}$ increased from 1.07 to a maximal value of 1.5, while the average risk $\bar{R_{0}}$ varied even less, from an initial value of 1.04 to a maximum of 1.23. In other words, the main effect of modifying the host community is in increasing the duration of the annual period of time with a risk of WNV outbreaks, rather than in amplifying the daily risk of such outbreak. Interestingly, the changes in the abundance of *Q*. *mexicanus* have larger impacts than the changes in the abundance of Columbiformes on all three quantities; P, $\bar{R_{0}}$ and $R_{0}^{max}$.

To further quantify these trends we performed an elasticity analysis where the effect of varying the abundance of *Q*. *mexicanus* (Columbiformes) is assessed conditionally to each of the abundance of Columbiformes (*Q*. *mexicanus*) (Fig 4D). The elasticity analysis clearly shows that the duration of the period at risk of WNV outbreaks (P) strongly depends on the change in the abundance of *Q*. *mexicanus* as a 1% increase in the latter sparks a 6.6% to 7.8% increase of duration, according to the abundance of Columbiformes. Meanwhile, a similar increase in the abundance of Columbiformes would only rise such a duration by 0.8% to 2.1%, according to the abundance of *Q*. *mexicanus*. On average, the period at risk of WNV outbreaks (P) was then found to be about 4.8 times more sensitive to changes in the abundance of *Q*. *mexicanus*. As described above, increasing the abundance of *Q*. *mexicanus* and Columbiformes has much lower effects, typically 10 times smaller, on the average ($\bar{R_{0}}$) and on the maximal ($R_{0}^{max}$) risks encountered during such a period. A 1% increase in the abundance of *Q*. *mexicanus* is predicted to rise $\bar{R_{0}}$ by 0.2% to 0.34% and $R_{0}^{max}$ by 0.48% to 0.72%, according to the abundance of Columbiformes, while the effects of a 1% increase in the abundance of Columbiformes is anticipated to increase $\bar{R_{0}}$ by 0.03% to 0.16% and $R_{0}^{max}$ by 0.05% to 0.27%, according to the abundance of *Q*. *mexicanus*. On average, the mean ($\bar{R_{0}}$) and maximal ($R_{0}^{max}$) risks of WNV outbreaks (P) were then found to be about 2.5 and 2.7 times more sensitive to changes in the abundance of *Q*. *mexicanus* than in the abundance of Columbiformes.

### Potential of vector control to limit the risk of WNV emergence

**Fig 5:**
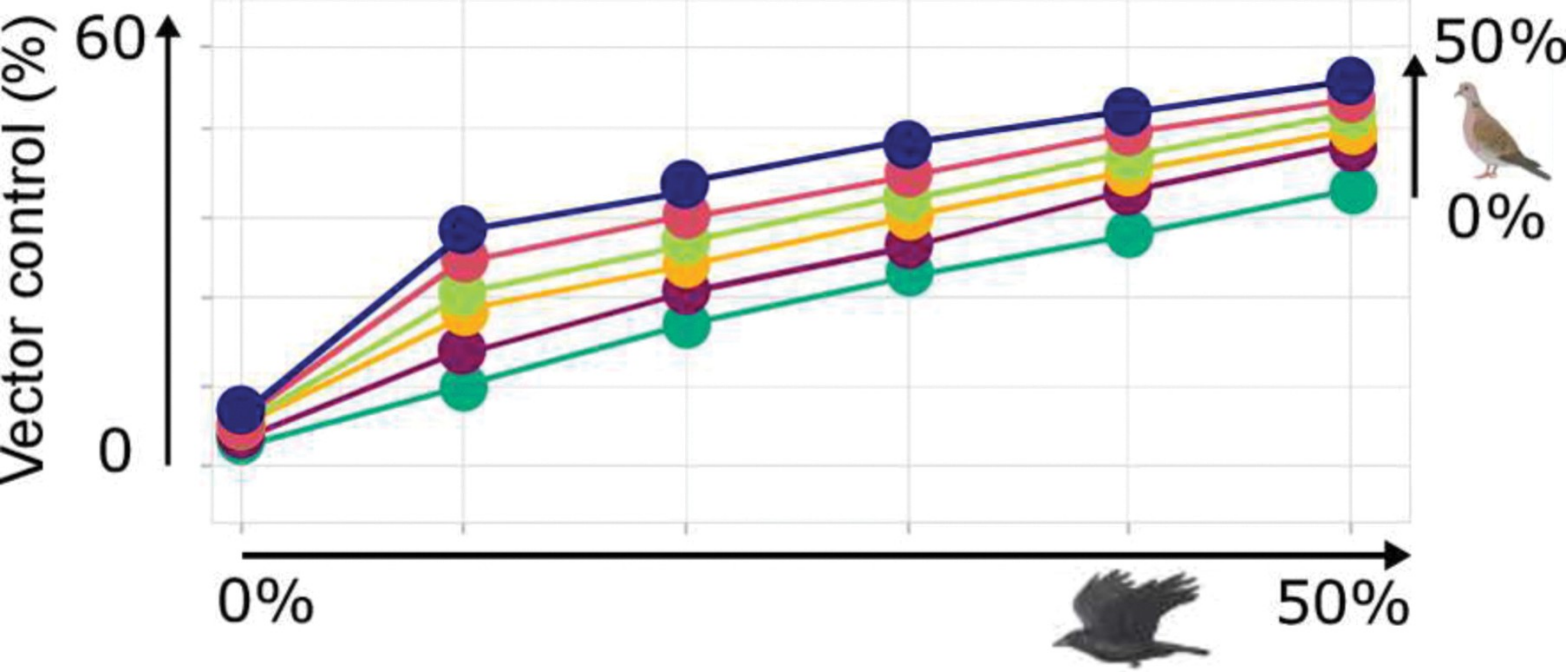
Potential of vector control to limit the risk of WNV outbreak in Merida, Mexico. The percentage of the vector population that shall be removed by vector control for R_0_ to remain lower than one all year round is 13% for the current bird community. The variations of this required percentage of vector control (y-axis) was estimated while varying the percentages of *Q*. *mexicanus* and of columbiform species. The frequency of *Q*. *mexicanus* in the bird community was increased from 0 to 50% (by 10%) along the x-axis, while the different colors stand for different frequency of columbiforms as follows: green = 0%, violet = 10%, yellow = 20%, light green = 30%, red = 40% and blue = 50%. Created with BioRender.com.

Our estimate of the current risk of WNV outbreak and our predictions about its future point towards calculating the fraction of the vector population that should be removed for R_0_ to remain lower than one all year round in the various contexts considered above (Fig 5). To reach that objective and annihilate the current risk of outbreaks, the size of the mosquito population should be reduced by ~13%. A 50% increase in the abundance of Columbiformes would only require to strengthen this effort to reach a ~17% reduction in vector abundance, but a similar increase in the abundance of *Q*. *mexicanus* would impose to limit the vector population size by up to 43%. Obviously, simultaneous rises in the abundance of the two taxa would make an even stronger control necessary, with an up to 56% reduction required to compensate for a 50% increase in *Q*. *mexicanus* and in Columbiformes.

## Discussion

Global urbanization is a defining trend of the Anthropocene [67] that favors the emergence of new pathogens [2,3] and rises the burden of infectious disease already afflicting human populations [4]. A large set of studies have provided evidence that the ongoing changes in urban areas favor WNV-competent mosquitoes ([23,24] and reference therein). While urbanization has also been shown to reduce bird biodiversity [25,26] and to increase the abundance of key species representing good reservoirs for the WNV [27], the contribution of such alteration of the bird community to the risk of WNV outbreaks remains much less investigated. To advance our quantitative understanding of risk of WNV outbreaks in developing cities in this direction, there is thus an obvious interest in implementing integrative models that bring together entomological, epidemiological and ornithological data [68–70].

### A seasonal risk of WNV outbreak in the city of Merida, and the need for epidemiological surveillance

We developed such an R0-modelling of the transmission of WNV by *C*. *quinquefasciatus* in the passeriform and columbiform community sampled in the city of Merida, Yucatan, Mexico. Our model predicts a strong seasonal pattern in the circulation of WNV amongst local birds with a summer peak associated to a 3-weeks period (starting around mid-July) when R_0_ exceeds 1, which is mostly explained by the low mosquito death rate and the high vector to host ratio encountered at that time of the year. The mosquito populations are then able to sustain and amplify WNV transmission in the bird community, strongly suggesting a time-limited but significant risk of outbreak of infections in humans. This prediction is consistent with previous field studies demonstrating that the virus is circulating in birds, horses and bats [34,71,72] in Merida, although there is no available record of infection in humans. Those empirical and modelling results outcomes clearly point toward the need to implement epidemiological surveillance in and out the city of Merida to better inform the circulation of WNV between animals and humans, and to provide the opportunity to design inclusive ‘One Health’ preventive strategies [73,74].

### The absence of large WNV outbreaks in humans, and the virus *vs* vector hypotheses

While the WNV has been introduced in Latin America and the Caribbean in the 2000’s [75,76], it has yet failed to produce severe human outbreaks in urban places [13] despite the virus being repeatedly reported in various bird or horse species, such as in Merida, Mexico [34], Brazil [77], Guatemala [78,79] and various other places in Latin America [80]. One of the most common hypotheses to explain such pattern is human protection by cross-immunity with other viral infections such as dengue, yellow fever and the Rocio or Saint-Louis encephalitis [13,80–83]. Alternative hypotheses, reviewed by [80] and [13], suggest that the lack of large outbreaks might be due to the dilution of WNV transmission by higher levels of biodiversity in the tropics, to under-reporting of cases because of asymptomatic infections [84], misdiagnosis by confusion of the symptoms with those of other diseases [76,85], cross-reactivity in serologic tests [86,87], and/or to the typical difficulty in monitoring public health in remote places such as the Amazonian region [13]. Recently, [13] emphasized that the absence of human cases of WNV encephalitis or severe disease in South American countries could be explained by the circulation of mutant strains with lower level of pathogenicity. Interestingly, our modelling showed that reducing the bird death from WNV (α) tends to increase (up to almost 4 times) the duration of the period of risk of WNV outbreaks and, to a lower extent (up to 1.4 times), the maximal and average R_0_ values of the virus during such period. Those effects are readily explained by the increase in the duration of the infectious period (equal to 1/(μ_j_+α)) that is associated with the reduction of the bird death from the disease (α). Accordingly, although strains with lower pathogenicity may indeed lead to asymptomatic cases and reduce apparent public health concerns [13], they also are spreading at larger rate according to a typical trade-off already documented for other human-pathogens, e.g. [88–91]. Our sensitivity analyses further showed that the risk of WNV outbreaks strongly rises with higher vector biting rates (a) and vector to host ratio (m), which is consistent with the conclusion of [92] who previously used a SIR model to calculate the R_0_ of WNV in a bird community, and identified vector (demographic and biting) parameters as key determinants of the rate of virus circulation. Those two quantitative studies therefore point toward an alternative working hypothesis that the absence of large outbreaks of WNV in Latin America could be caused by a limited transmission due to vectors’ features rather than by the virus life-history.

### Prevention strategies by vector and bird control

Among the three parameters that were identified as having the largest impact on the duration and intensity of the risk of WNV outbreaks (see discussion above), only the vector to host ratio (m) can be targeted through appropriate control strategies. In order to reduce *m and lower WNV transmission among urban birds*, *typical vector control* strategies could be implemented to limit *C*. *quinquefasciatus* abundance. As expected, the level of preventive vector control required to annihilate the predicted risk of outbreaks in human, i.e. for R_0_ to be lower than 1 all year round, varies with the structure of the bird community. According to our model predictions, a reduction of ~13% of the *C*. *quinquefasciatus* population would be enough to reach such a public health objective in the city of Merida with its current columbiform and passeriform community. However, such a relatively low figure may be deceptive as our modelling also shows that it could rise up to 17%, 43% and 56%, if the abundance of Columbiformes, Passeriformes or both taxa were to increase. While preventive strategies targeting *C*. *quinquefasciatus* could probably be implemented at somewhat reduced costs by adequately integrating them into existing vector control program in Merida [93], the last predictions strongly suggest that concomitantly controlling the bird population dynamics might substantially help by keeping low the target for vector control. In such perspectives, another key outcome of our modelling is that bird taxa make significantly different contributions to the overall risk of transmission, with *Q*. *mexicanus emerging as* the host with the largest impact on WNV transmission, as previously reported in the city of Puerto Barrios, Guatemala [79]. The mean and maximal risks of WNV outbreaks were indeed found to be 2.5 to 2.7 times more sensitive to changes in the abundance of *Q*. *mexicanus* than in the abundance of Columbiformes. This highly competent species thus seems to play a similar role of superspreader as *Turdus migratorious* in New York, USA [94], which suggests that it would be worth to (at least) monitor the growth of its population and survey its prevalence of infection by the WNV infectious status while the city of Merida is further developing. Such urban field studies might actually be beneficial to our understanding of other public health concerns in the Americas. The generalist behavior and ability to adapt to urban environment of *Q*. *mexicanus* has indeed contributed to its spread from Central to North [95] and South [96] America, and as made it a good reservoir of other human pathogens such as *Salmonella spp*. [97], *Sarcocystis sp*. [98], trypanosomes, haemosporida, and filarial nematodes co-circulating in Texas, USA [99].

To conclude, this study highlights the potential for WNV outbreak of human infection in the urban Merida, Mexico, and identifies *Q*. *mexicanus* as a local superspreader of the virus. This naturally suggests to implement a dedicated epidemiological monitoring and preemptive measures concomitantly targeting *C*. *quinquefasciatus* and *Q*. *mexicanus* populations, as those two control strategies are expected to have synergistic effects.

## Supporting information

S1 Appendix List of all passeriform and columbiform species identified in Merida, Mexico, with the estimates of their abundance, lifespan, competence for WNV and of the duration of WNV viremia. (PDF)

S2 Appendix Sensitivity analyses of the seasonal variations in WN-RM_0_ with respect to a recovery rate of birds. (PDF)

S3 Appendix Sensitivity analyses of the intensity and duration of the risk of WNV outbreak in Merida, Mexico. (PDF)
