# Supplementary material for: Assessing the risk of West Nile Virus seasonal outbreaks and its vector control in an urbanizing bird community: An integrative R0-modelling study in the city of Merida, Mexico
Source: PLoS Negl Trop Dis. 2023 May 30;17(5):e0011340. doi: 10.1371/journal.pntd.0011340 (PMC10256229; doi:10.1371/journal.pntd.0011340)
Supplement: S1 Appendix — (PDF) [file pntd.0011340.s001.pdf]

Species	Family	Abundance [a, b]	Lifespan [c] (years)	Competences [d] C	Duration of viremia [e,f] D (days)	Group of species
**Passeriforms**						
Cyanocorax yncas	Corvidae	37 (0.71)	10.7 [1]	1.66	4.6 (e1)	P1
Cyanocorax yucatanicus	Corvidae	200 (3.84)	10.7 ([1],c1)	1.66	4.6 (e1)	P1
Arremonos rufivirgatus	Emberizidae	6 (0.11)	6.9 [2]	1.41 (d1)	6.0 (e2)	P2
Passerina cyanea	Cardinalidae	111 (2.13)	11.0 [1]	1.41 (d1)	6.0 (e2)	P2
Pheucticus ludovicianus	Cardinalidae	12 (0.23)	7.3 [1]	1.41 (d1)	6.0 (e2)	P2
Piranga rubra	Cardinalidae	38 (0.73)	7.3 [1]	1.41 (d1)	6.0 (e2)	P2
Piranga olivacea	Cardinalidae	1 (0.02)	10.1 [1]	1.41 (d1)	6.0 (e2)	P2
Euphonia affinis	Fringillidae	15 (0.29)	9.0 ([1], c2)	1.41 (d1)	6.0 (e2)	P2
Spinus psaltria	Fringillidae	16 (0.31)	5.7 ([3], c3)	1.41 (d1)	6.0 (e2)	P2
Dives dives	Icteridae	179 (3.43)	10.9 ([1],c4)	1.19	3.15	P2
Icterus auratus	Icteridae	62 (1.19)	8.6 ([1],c5)	1.19	3.15	P2
Icterus cucullatus	Icteridae	16 (0.31)	6.0 [1]	1.19	3.15	P2
Icterus gularis	Icteridae	165 (3.16)	8.6 ([1],c5)	1.19	3.15	P2
Icterus spurius	Icteridae	135 (2.59)	9.6 [1]	1.19	3.15	P2
Molothrus aeneus	Icteridae	45 (0.86)	3.6 [1]	1.19	3.15	P2
Quiscalus mexicanus	Icteridae	2082 (39.91)	12.5 [1]	1.28; 2.01 [f]	2.01; 0.45 [f]	Q
Dumetella carolinensis	Mimidae	11 (0.21)	10.9 [4]	0.30 (d2)	3.2 (e3)	P3
Mimus gilvus	Mimidae	246 (4.71)	20.0 ([1], c6)	0.30 (d2)	3.2 (e3)	P3
Euphonia trichas	Parulidae	55 (1.05)	6.9 ([1], c7)	1.19 (d3)	3.15	P2
Mniotilta varia	Parulidae	16 (0.31)	11.3 [1]	1.19 (d3)	3.15	P2
Oreothypis peregrina	Parulidae	59 (1.13)	6.9 ([1], c7)	1.19 (d3)	3.15	P2
Parkesia noveboracensis	Parulidae	22 (0.42)	8.9 ([3], c8)	1.19 (d3)	3.15	P2
Protonotaria citrea	Parulidae	1 (0.02)	4.9 [1]	1.19 (d3)	3.15	P2
Seiurus aurocapilla	Parulidae	2 (0.04)	9.0 [1]	1.19 (d3)	3.15	P2
Setophaga americana	Parulidae	25 (0.48)	8.6 ([3], c9)	1.19 (d3)	3.15	P2
Setophaga citrina	Parulidae	16 (0.30)	8.2 [3]	1.19 (d3)	3.15	P2

Setophaga dominica	Parulidae	23 (0.44)	6.1 [3]	1.19 (d3)	3.15	P2
Setophaga fusca	Parulidae	3 (0.06)	8.2 [3]	1.19 (d3)	3.15	P2
Setophaga magnolia	Parulidae	9 (0.17)	8.6 ([3], c9)	1.19 (d3)	3.15	P2
Setophaga palmarum	Parulidae	1 (0.02)	6.6 [4]	1.19 (d3)	3.15	P2
Setophaga petechia	Parulidae	43 (0.82)	11.0 [4]	1.19 (d3)	3.15	P2
Setophaga ruticilla	Parulidae	5 (0.09)	10.1 [4]	1.19 (d3)	3.15	P2
Setophaga virens	Parulidae	12 (0.23)	6.0 [4]	1.19 (d3)	3.15	P2
Polioptila caerulea	Polioptilidae	308 (5.90)	4.2 [1]	0.30 (d2)	3.2 (e3)	P3
Saltator coerulescens	Thraupidae	151 (2.90)	7.7 ([1], c10)	1.19 (d3)	3.15	P2
Thraupis episcopus	Thraupidae	21 (0.40)	9.0 [1]	1.19 (d3)	3.15	P2
Tiaris olivaceus	Thraupidae	3 (0.06)	7.7 ([1], c10)	1.19 (d3)	3.15	P2
Volatinia jacarina	Thraupidae	4 (0.08)	7.7 ([1], c10)	1.19 (d3)	3.15	P2
Thryothorus ludovicianus	Troglodytidae	14 (0.27)	6.1 [1]	0.30 (d2)	3.2 (e3)	P3
Troglodytes aedon	Troglodytidae	39 (0.75)	7.1 [1]	0.30 (d2)	3.2 (e3)	P3
Uropsila leucogastra	Troglodytidae	2 (0.04)	6.3 ([1], c11)	0.30 (d2)	3.2 (e3)	P3
Catharus ustulatus	Turdidae	1 (0.02)	11.0 [1]	0.10	1.0	P3
Turdus grayi	Turdidae	233 (4.47)	9.6 [3]	0.10	1.0	P3
Contopus cinereus	Tyrannidae	7 (0.13)	6.8 ([1], c12)	1.66 (d4)	4.6 (e4)	P1
Contopus virens	Tyrannidae	2 (0.04)	7.1 [1]	1.66 (d4)	4.6 (e4)	P1
Empidonax minimus	Tyrannidae	40 (0.77)	10.9 [1]	1.66 (d4)	4.6 (e4)	P1
Megarynchus pitangua	Tyrannidae	2 (0.04)	6.0 [1]	1.66 (d4)	4.6 (e4)	P1
Myiarchus tuberculifer	Tyrannidae	26 (0.5)	10.9 ([1], c13)	1.66 (d4)	4.6 (e4)	P1
Myiarchus tyrannulus	Tyrannidae	13 (0.25)	9.9 [1]	1.66 (d4)	4.6 (e4)	P1
Myiarchus yucatanensis	Tyrannidae	4 (0.08)	10.9 ([1], c13)	1.66 (d4)	4.6 (e4)	P1
Myiozetetes similis	Tyrannidae	155 (2.97)	7.2 ([1], c14)	1.66 (d4)	4.6 (e4)	P1
Pitangus sulphuratus	Tyrannidae	161 (3.09)	6.9 [4]	1.66 (d4)	4.6 (e4)	P1
Tyrannus couchii	Tyrannidae	9 (0.17)	8.4 ([1], c15)	1.66 (d4)	4.6 (e4)	P1
Tyrannus melancholicus	Tyrannidae	114 (2.19)	8.4 ([1], c15)	1.66 (d4)	4.6 (e4)	P1
Tyrannus tyrannus	Tyrannidae	13 (0.25)	9.9 [1]	1.66 (d4)	4.6 (e4)	P1

Cyclarhis gujanensis	Vireonidae	106 (2.03)	8.1 ([1], c16)	1.66 (d4)	4.6 (e4)	P1
Vireo flavifrons	Vireonidae	6 (0.12)	6.1 [1]	1.66 (d4)	4.6 (e4)	P1
Vireo griseus	Vireonidae	91 (1.74)	7.3 [1]	1.66 (d4)	4.6 (e4)	P1
Vireo olivaceus	Vireonidae	1 (0.02)	10.0 [1]	1.66 (d4)	4.6 (e4)	P1
Vireo pallens	Vireonidae	21 (0.40)	7.9 ([1], c16)	1.66 (d4)	4.6 (e4)	P1
**Columbiforms**						
Columba livia	Columbidae	267 (9.58)	6.3 [4]	0.19	3.3	Co
Columbina talpacoti	Columbidae	178 (6.38)	7.5 [4]	0.19	3.3	Co
Leptotila verreauxi	Columbidae	50 (1.79)	8.6 [4]	0.19	3.3	Co
Patagioenas flavirostris	Columbidae	1 (0.04)	18 [4]	0.19	3.3	Co
Streptopelia decaocto	Columbidae	157 (5.63)	13.7 [4]	0.19	3.3	Co
Zenaida asiatica	Columbidae	2135 (76.58)	21.8 [4]	0.19	3.3	Co

(a) The abundances are given both in absolute densities and as percentages of the total Passeriforms or Columbiforms abundances.

(b) All abundance data were collected in Vidal (2016) and reference 5 for the Columbiforms species *Columba livia* and *Zenaida asiatica*.

(c) Estimates of lifespan were found in references 1 to 5 (see below). c1 = estimate made for the close species Cyanocorax yncas, c2 = average of estimates made for 22 Parulidae species, c3 = estimate made for the close species Stelgidopteryx ruficollis, c4 = average of estimates made for 19 Icteridaes species, c5 = average of estimates made for 6 Icteridaes species, c6 = estimate made for the close species Mimus polyglottos, c7 = average of estimates made for Fringillidae species, c8 = estimate made for the close species Seiurus noveboracensis, c9 = average of estimates made for 19 Setophaga species, c10 = average of estimates made for 3 Thraupidae species, c11 = average of estimates made for 5 Troglodytidae species, c12 = average of estimates made for 4 Contopus species, c13 = average of estimates made for 3 Myiarchus species, c14 = average of estimates for 22 Tyrannidae species, c15 = average of estimates made for 2 Tyrannus species, c16 = average of estimates made for 10 Viero species.

(d) Estimates of reservoir competence index were found in references 6 to 8 (see below). The estimates of C were typically available at the family level. For each species, we then used the value estimated for the closest phylogenetic family. d1 = estimate for Fringillidae, d2 = estimate for Sturnidae, d3 = estimate for Icteridae, d4 = estimate for Corvidae.

(e) Estimates of the duration of viremia were found in references 6 to 8 (see below). e1 = average of estimates made for 3 Corvidae species, e2 = estimate made for the close species *Carpodacus mexicanus*, e3 = average of estimates made for 2 Iceteridae species, e4 = average of estimates made for 3 Corvidae species.

(f) Two sets of estimates of C and D were available for *Quiscalus mexicanus* in reference 7, so that we derived two estimates of the desired quantitites bc (where b and c stand for the per bite probabilities of virus transmission from vector to host and from host to vector, respectively) from the ratio C/D (as explained in the main text), and considered the average of these two estimates.

Reference

[1] Wasser DE, Sherman PW. Avian longevities and their interpretation under evolutionary theories of senescence. Journal of Zoology. 2010;280(2):103–55.
[2] World Life Expectancy [Internet]. World Life Expectancy. [Acceded in 2021 Jun]. Available from: https://www.worldlifeexpectancy.com/
[3] Human Ageing Genomic Resources [Internet]. [Acceded in 2021 Jun]. Available from: https://genomics.senescence.info/
[4] ADW: Home [Internet]. [cited 2022 Nov 15]. Available from: https://animaldiversity.org/
[5] CONABIO. Aves MX [Internet]. [cited 2022 Nov 15]. Available from: http://avesmx.conabio.gob.mx/
[6] Komar N, Langevin S, Hinten S, Nemeth N, Edwards E, Hettler D, et al. Experimental Infection of North American Birds with the New York 1999 Strain of West Nile Virus. Emerg Infect Dis. 2003 Mar;9(3):311–22.
[7] Kilpatrick AM, LaDeau S, Marra PP. ECOLOGY OF WEST NILE VIRUS TRANSMISSION AND ITS IMPACT ON BIRDS IN THE WESTERN HEMISPHERE. The Auk. 2007;124(4):1121–236.
[8] Guerrero-Sánchez S, Cuevas-Romero S, Nemeth NM, Trujillo-Olivera MTJ, Worwa G, Dupuis A, et al. West Nile Virus Infection of Birds, Mexico. Emerg Infect Dis. 2011 Dec;17(12):2245–52.
