# Supplementary material for: Assessing the risk of West Nile Virus seasonal outbreaks and its vector control in an urbanizing bird community: An integrative R0-modelling study in the city of Merida, Mexico
Source: PLoS Negl Trop Dis. 2023 May 30;17(5):e0011340. doi: 10.1371/journal.pntd.0011340 (PMC10256229; doi:10.1371/journal.pntd.0011340)
Supplement: S2 Appendix — (PDF) [file pntd.0011340.s002.pdf]

In this appendix, we aim at including a recovery of birds so that an infected host can either die from the infection, with probability p, or recover, with probability 1-p. In the former case, the infectious life time is given by $1/(\mu_j+\alpha)$ (as in equations 2 and 4 of the main text), while in the latter it equals $1/(\mu_j+\gamma)$ where γ stands for the recovery rate. This leads to the following change in the expression of the mean number of vectors infected by one infected host of group j during its infectious lifetime :

$$k_{Vj} = p\left(\frac{mp_j ac_j}{(\mu_j+\alpha)}\right) + (1-p)\left(\frac{mp_j ac_j}{(\mu_j+\gamma)}\right) \qquad \textbf{Eq. 2'}$$

The R_0 for the transmission of WNV by 1 vector to N groups of bird species can then be re-calculated as the dominant eigenvalue of the NGM given by equation 3 from the main text, which can then be expressed as:

$$R_0 = \sqrt{\sum_{j=1}^{N} k_{jV} k_{Vj}} = \sqrt{\sum_{j=1}^{N} \frac{(ap_j)^2 mbc_j\,\kappa}{\mu_V(\kappa+\mu_V)}\left(\frac{p}{\mu_j+\alpha} + \frac{1-p}{(\mu_j+\gamma)}\right)} \qquad \textbf{Eq. 4'}$$

Using equation 4' we predicted the monthly variations of R_0 shown in figure 2, while p and γ took on values estimated from the literature. According to [1], an average proportion of $p\sim0.59$ (13/22) of inoculated birds died of infection, and according to estimates of recovery rates found in [2] whose average was calculated to be equal to $\gamma\sim0.27$. The seasonal variations of R_0 in the presence of such recovery were strictly similar to what was predicted in their absence in Figure 2, and the maximal value of R_0 (R_0^{max}), the duration of the period at risk of outbreak (P) and the average value of R_0 during that period ($\overline{R_0}$) all changed only by -1.4%, -13% and -0.8%, respectively. The robustness of the prediction to the existence of recovery is easily explained. The rates α and γ being very similar, i.e. 0.25 *.vs.* 0.27, and much larger than μ_j's, whatever the proportions of individuals dying/recovering, the average duration of the infectious period would remain similar. Actually, the maximal variations that could be obtained can be estimated by considering the differences in predictions when p=1 (the case implicitely considered in the main text) and when p=0 (when no birds die and they all recover). This leads to a maximal change in R_0^{max}, P and $\overline{R_0}$ of -3.6%, -17% and -1.9%, respectively.

Reference

[1] Guerrero-Sánchez S, Cuevas-Romero S, Nemeth NM, Trujillo-Olivera MTJ, Worwa G, Dupuis A, et al. West Nile Virus Infection of Birds, Mexico. Emerg Infect Dis. 2011 Dec;17(12):2245–52.

[2] Cruz-Pacheco G, Esteva L, Montano-Hirose J, Vargas C. Modelling the dynamics of West Nile Virus. Bulletin of Mathematical Biology. 2005 Nov;67(6):1157–72.
