# Supplementary material for: Assessing the risk of West Nile Virus seasonal outbreaks and its vector control in an urbanizing bird community: An integrative R0-modelling study in the city of Merida, Mexico
Source: PLoS Negl Trop Dis. 2023 May 30;17(5):e0011340. doi: 10.1371/journal.pntd.0011340 (PMC10256229; doi:10.1371/journal.pntd.0011340)
Supplement: S3 Appendix — (PDF) [file pntd.0011340.s003.pdf]

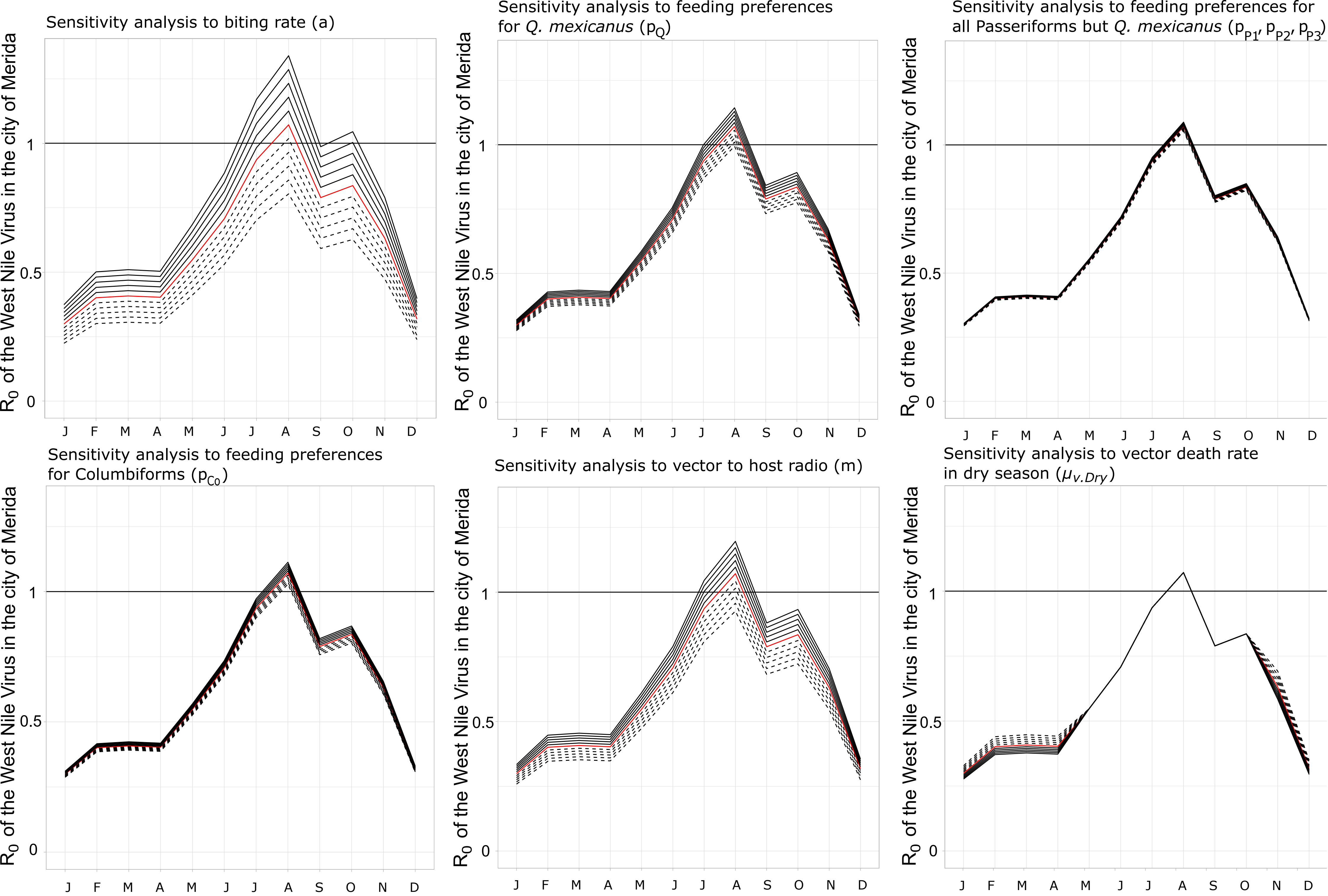
Sensitivity analysis to biting rate (a)
Sensitivity analysis to feeding preferences for Q. mexicanus ($p_Q$)
Sensitivity analysis to feeding preferences for all Passeriforms but Q. mexicanus ($p_{P1}$, $p_{P2}$, $p_{P3}$)
Sensitivity analysis to feeding preferences for Columbiforms ($p_{Co}$)
Sensitivity analysis to vector to host radio (m)
Sensitivity analysis to vector death rate in dry season ($\mu_{v.Dry}$)
$R_0$ of the West Nile Virus in the city of Merida
0
0.5
1
J F M A M J J A S O N D

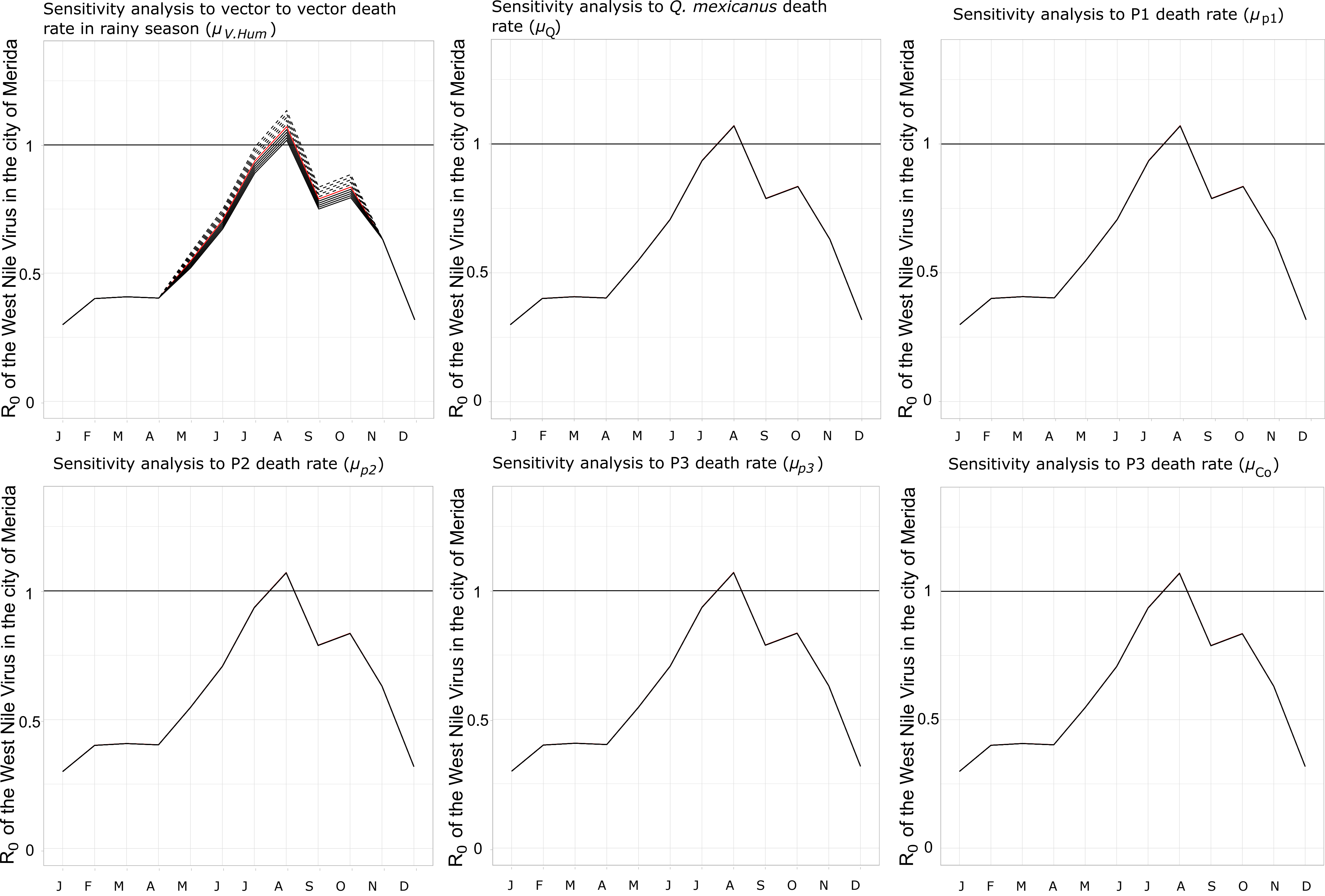

Sensitivity analysis to vector to vector death rate in rainy season ($\mu_{V.Hum}$)
Sensitivity analysis to *Q. mexicanus* death rate ($\mu_Q$)
Sensitivity analysis to P1 death rate ($\mu_{p1}$)
Sensitivity analysis to P2 death rate ($\mu_{p2}$)
Sensitivity analysis to P3 death rate ($\mu_{p3}$)
Sensitivity analysis to P3 death rate ($\mu_{Co}$)
$R_0$ of the West Nile Virus in the city of Merida
0
0.5
1
J F M A M J J A S O N D

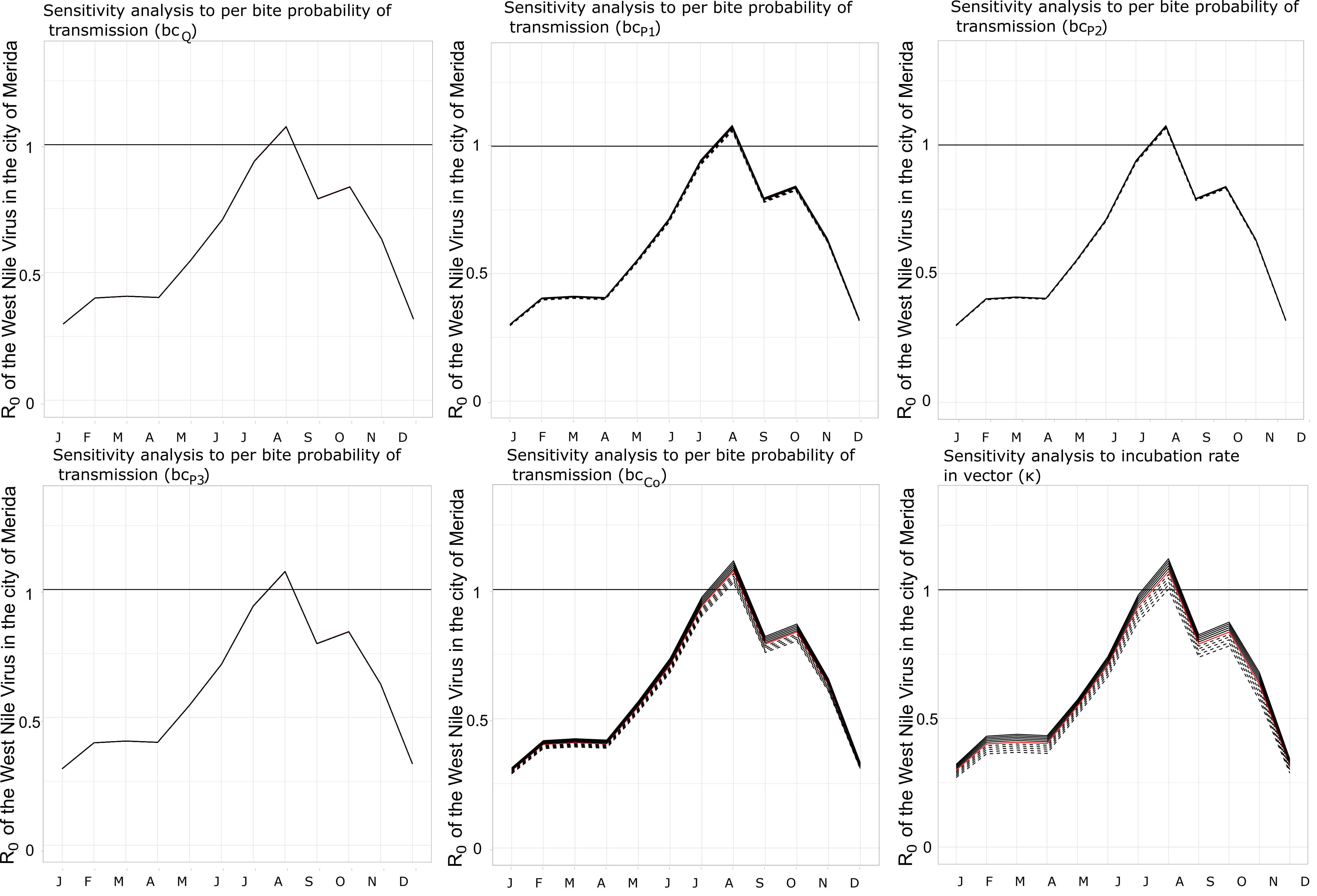

Sensitivity analysis to per bite probability of transmission ($bc_Q$)
Sensitivity analysis to per bite probability of transmission ($bc_{P1}$)
Sensitivity analysis to per bite probability of transmission ($bc_{P2}$)
Sensitivity analysis to per bite probability of transmission ($bc_{P3}$)
Sensitivity analysis to per bite probability of transmission ($bc_{Co}$)
Sensitivity analysis to incubation rate in vector (κ)
$R_0$ of the West Nile Virus in the city of Merida
0
0.5
1
J F M A M J J A S O N D

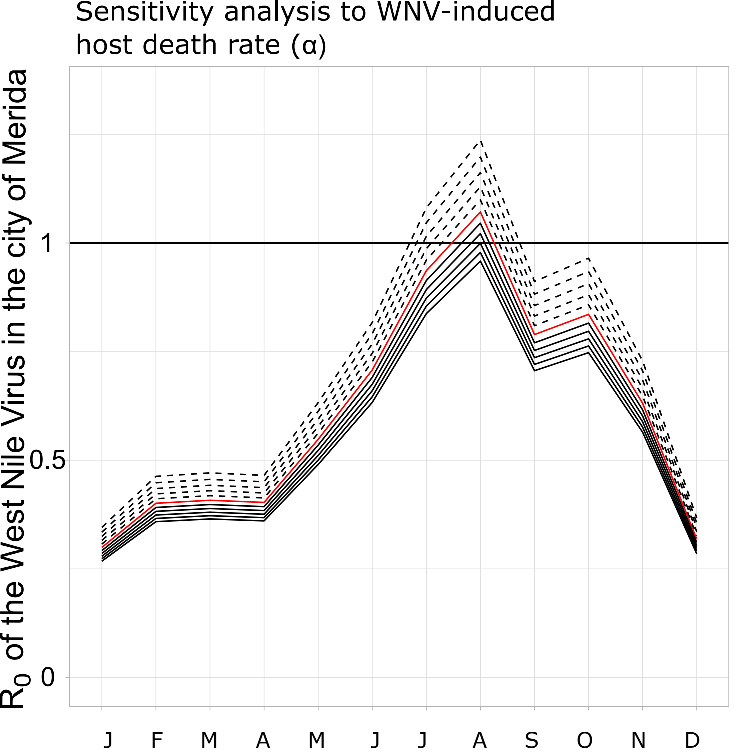
Sensitivity analysis to WNV-induced
host death rate (α)
R0 of the West Nile Virus in the city of Merida
1
0.5
0
J
F
M
A
M
J
J
A
S
O
N
D
